# Protein Receptors Evolved from Homologous Cohesion Modules That Self-Associated and Are Encoded by Interactive Networked Genes

**DOI:** 10.3390/life11121335

**Published:** 2021-12-03

**Authors:** Donard S. Dwyer

**Affiliations:** Departments of Psychiatry and Behavioral Medicine and Pharmacology, Toxicology and Neuroscience, LSU Health Shreveport, 1501 Kings Highway, Shreveport, LA 71130, USA; donard.dwyer@lsuhs.edu

**Keywords:** cohesion modules, gene interaction networks, receptor evolution, self-organization, syntenic blocks of genes

## Abstract

Previously, it was proposed that protein receptors evolved from self-binding peptides that were encoded by self-interacting gene segments (inverted repeats) widely dispersed in the genome. In addition, self-association of the peptides was thought to be mediated by regions of amino acid sequence similarity. To extend these ideas, special features of receptors have been explored, such as their degree of homology to other proteins, and the arrangement of their genes for clues about their evolutionary origins and dynamics in the genome. As predicted, BLASTP searches for homologous proteins detected a greater number of unique hits for queries with receptor sequences than for sequences of randomly-selected, non-receptor proteins. This suggested that the building blocks (cohesion modules) for receptors were duplicated, dispersed, and maintained in the genome, due to structure/function relationships discussed here. Furthermore, the genes coding for a representative panel of receptors participated in a larger number of gene–gene interactions than for randomly-selected genes. This could conceivably reflect a greater evolutionary conservation of the receptor genes, with their more extensive integration into networks along with inherent properties of the genes themselves. In support of the latter possibility, some receptor genes were located in active areas of adaptive gene relocation/amalgamation to form functional blocks of related genes. It is suggested that adaptive relocation might allow for their joint regulation by common promoters and enhancers, and affect local chromatin structural domains to facilitate or repress gene expression. Speculation is included about the nature of the coordinated communication between receptors and the genes that encode them.

## 1. Introduction

**Figure 1:**
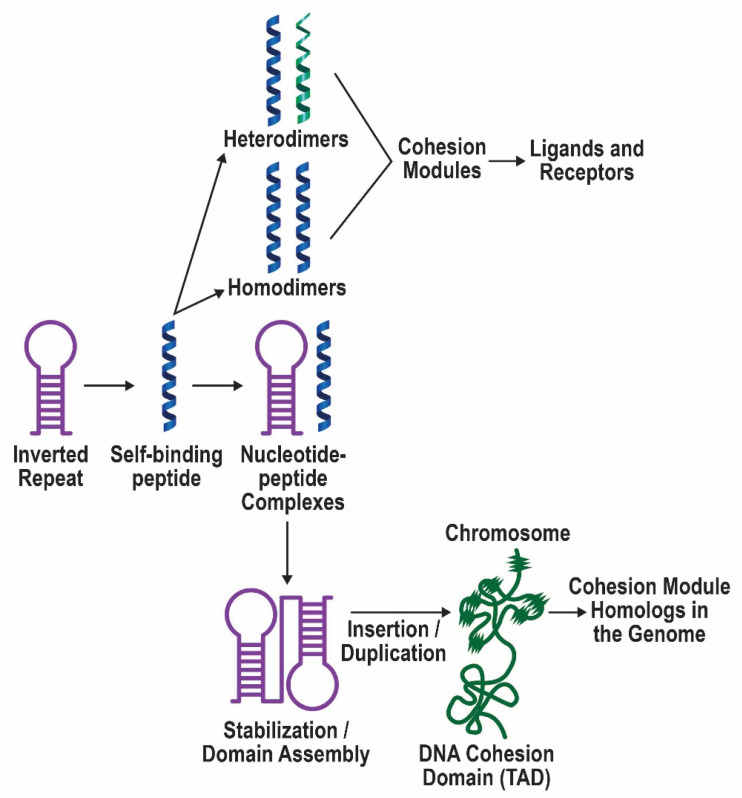
Depiction of the self-associating peptide model of the evolution of protein ligand–receptor pairs. Inverted repeats encoded self-binding peptides that formed homodimers and eventually heterodimers. Evidence for this part of the model has been presented previously [3]. These self-binding peptides/cohesion modules provided the building blocks for ligands and receptors. When the peptides also bound to the sequence that encoded them, this interaction promoted the survival of that sequence. Such inverted repeats were favored for insertion, duplication, and merging to produce more complex domain structures. While gene duplications often remain in the vicinity of the original gene, they may also disperse in the genome, as has been observed for large superfamilies of receptor proteins. Homologous regions in the genome are indicated by wavy lines on the chromosome and comprise cohesion modules in receptor proteins and diverged ancestors. Based on retained similarities, these special regions of DNA may interact within and between chromosomes giving rise to gene–gene interactions and topologically associated domains (TADs). Furthermore, distinctive features of the replicated DNA sequences may have facilitated adaptive gene relocation, which, during evolution, brought together genes that function in the same biological process. It is speculated that the creation of these syntenic blocks of genes may have then allowed their joint regulation by enhancers/promoters, and coordinated their expression via shared chromatin accessibility.

Ligand–receptor interactions are essential for life and mediate intercellular communication, hormone signaling, neurotransmission, immune function, metabolic processes, and more. The co-evolution of the molecules involved is a fascinating story that is still unfolding [1,2,3,4,5,6]. Although various theories exist to explain how peptide/protein ligands and their protein receptors have evolved, two have received more experimental support: the self-associating peptide theory [1,3] and the molecular complementarity model [4,7]. According to the self-associating peptide theory, self-binding peptides were encoded in the proto-genome by self-binding nucleotide sequences (inverted repeats) that were mobile (transposable) and extensively duplicated (see Figure 1). These transposable exons assembled into more complex, modular protein structures that eventually diverged and evolved into ligands and receptors that retained and refined the initial self-affinity of their ancestral peptides. Although ligand–receptor interactions may have originally been based on areas of amino acid sequence homology (dimer to ‘heterodimer’ transition [1]), this system was likely adapted to include complementarity as an additional mode of recognition, as elegantly proposed by Root-Bernstein [7]. Experimental evidence supports both of these models [7,8].

Ligand–receptor binding is a more evolved and specialized form of protein–protein interactions that include dimerization, heterodimerization, and subunit assembly into complex macromolecules [9,10]. Broadly conceptualized, these types of protein–protein interactions mediate molecular cohesion—the aggregation of similar molecules (like binds like [1]) in order to survive destructive forces such as hydrolysis, proteolysis, and denaturation. Here, I introduce the term cohesion modules for those regions of proteins that stabilize protein–protein interactions, whether the actual binding is mediated via homologous or complementary segments. During evolution, cohesion modules likely assumed a primary role in the self-assembly and organization of multi-subunit proteins, due to their dual role as promiscuous ‘sticky’ regions, and as insulators against degradation/denaturation. Consequently, there is great interest in being able to predict protein–protein interaction sites and to characterize the various domains (e.g., SH3, PDZ, EGF, and immunoglobulin-based modules) that stabilize proteins and create functional networks [11,12,13].

A close functional relationship between genes and proteins was probably established early on, when nucleotide polymers became proto-genes and amino acid/peptide condensation formed proteins. Previously, it was suggested that peptides that were encoded by early inverted repeats could provide additional protection to these segments of the proto-genome by binding to them [3], and there is evidence for this prediction [14]. Blalock and Smith [15] observed that codons and anti-codons specified amino acids with complementary physical properties that can mediate peptide/protein interactions [15,16], which provides a potential mechanism for the ancient relationship between genes and proteins. The DNA that encoded the building blocks of proteins, namely transposable exons, or trexons, was mobile, highly duplicated, and dynamic. Due to the fact that the genome was constructed from trexons [3], we would expect to still find potential evidence for the insertion of transposable elements, double strand break sites (due to the torsional stress of inverted repeat regions), and recombination hotspots. These features of the former trexon segments would have allowed for greater subsequent mutation, duplication, and divergence of sequences, which is consistent with the evolution of large receptor families such G protein-coupled receptors (GPCRs), immunoglobulins, and cytokine receptors [17,18,19]. Therefore, it seems to be an inescapable conclusion that peptide/protein receptors co-evolved with the DNA that encoded them, leading to the possibility of cohesion domains in the genome as well, i.e., regions of DNA that interact to ensure stability and allow the joint regulation of gene expression [20,21].

Based on these ideas about receptor evolution, several predictions follow. First, receptor proteins should exhibit greater amino acid sequence homology with other proteins in the genome when compared with non-receptor proteins. Secondly, the genes encoding receptor proteins should engage in more gene–gene interactions than genes for non-receptor proteins reflecting the process of evolution (e.g., greater gene duplication). Thirdly, receptor genes may have been adaptively relocated (gene amalgamation) to form functional blocks of genes that allow for the joint regulation of expression. Here, I evaluated these predictions and confirmed the special nature of receptors and their corresponding genes. Cohesion modules that are encoded by highly interactive genes with functional rearrangements in the genome provide new insights into the evolution of receptor–protein complexes.

## 2. Methods

### 2.1. Compilation of Receptor and Non-Receptor Gene Lists

The choice of receptor classes was intentional and included representatives of GPCRs, cytokine receptors, adhesion molecules, and hormone receptors, while excluding examples of immunoglobulins, T-cell receptors, major histocompatibility antigens, and odorant receptors, due to the large sizes of these families. Our goal was to examine a select subset of receptor genes that were representative of the different families of receptors such as cell adhesion molecules, GPCRs, and hormone and cytokine receptors. The selection of particular receptors within a representative class was random, and a total of 30 genes were chosen for the analysis. For comparison, a list of 30 randomly-selected human genes was created with the Random Gene Set Generator from Molbiotools (www.molbiotools.com). Obvious receptor genes were removed from the list and replaced with the next random non-receptor gene. As a final criterion for inclusion in the study, all genes from both the receptor and non-receptor candidate sets were first evaluated in GeneMANIA to ensure that they participated in genetic interactions with other genes in that dataset. Genes that failed to show any interactions in GeneMANIA were excluded from the sets. The lists of genes used in this study are summarized in Supplementary Table S1.

I chose to focus on a select, but representative set of receptor genes for two reasons. First, the large number of receptor genes in several major families of proteins (e.g., immunoglobulins, HLA proteins, and GPCRs, accounting for >15% of the entire genome) precluded a whole genome analysis. The large number of hits that would have been obtained with their inclusion in a genome-wide study would strongly bias the results in favor of receptors over non-receptors at the outset. Second, by restricting focus to a representative sample, it was possible to follow up with a more detailed analysis of local gene regions for evidence of genetic interactions and adaptive gene relocation (to form syntenic blocks). This analysis would reveal whether the genes coding for interactive cohesion modules were likewise special, with respect to their capability for interactions.

### 2.2. Analysis of Amino Acid Sequence Homology in Whole Genome Searches

The goal was to determine how many proteins were found in the entire human genome with defined regions of homology to receptor vs. non-receptor proteins. For this analysis, I used BLASTP at the Ensembl [22] website, with amino acid sequences which were derived from the corresponding human genes. The query sequences from the receptor and non-receptor lists were loaded, and the same search parameters were used throughout the study: distant homologies; *E* value 1.0; with 250 hits selected. The resultant lists were inspected manually and the number of unique hits was tallied for each protein. The homologous proteins are not considered to be orthologs of the query protein, but they show defined levels of sequence similarity used for the comparisons. This study focused on finding distant homologs of receptor sequences and was not aimed at finding protein–protein interactions, per se.

To fully characterize the local receptor–gene environment, it was necessary to explore counterparts of the human genes in lower species to detect gene rearrangements that occurred during evolution. In these instances, the human amino acid sequence for a receptor or flanking gene product was used to search for bona fide orthologs in other species, using BLASTP. The settings were similar to those used above; however, orthologs were confirmed by the detection of genes already notated as such in a database, or if they met the following criteria: *E* < 0.00001, a similar sequence length to the human protein, and the presence of syntenic markers (other established genes in similar relative locations).

### 2.3. Characterization of Receptor Gene Environment in the Genome

Previous work has identified syntenic blocks of genes that were apparently rearranged during evolution to be in close proximity, and which serve a common biological function [23,24]. To determine if any of the receptor genes experienced similar rearrangements into blocks or clusters, nearby genes were examined for their relationships with the receptor gene that included receptor-related activity or their involvement in similar or antagonistic biological functions. A number of regions qualified as potential syntenic blocks of genes. Relocated gene clusters such as these are a relatively new finding and, consequently, only a few have been described at length in the literature thus far [23,24,25,26,27,28,29,30]. Therefore, the novelty of this area of research limited the number of genes that could be analyzed in this study. To broaden this analysis, I included 5 established examples of syntenic blocks of genes which were identified previously [24] that contained receptor genes. Besides the receptor gene in the various blocks, 2 additional nearby genes defined the subset for analysis (see Supplementary Table S2). The 3-gene sets from each block were analyzed with GeneMANIA (next section) to determine if they formed a gene interaction network. In view of the small number of genes being examined, GeneMANIA was allowed to add up to 5 additional genes to construct the gene networks. If all 3 genes were included in the resulting network, that block received a score of 3, whereas if 2 of the genes interacted, a score of 2 was given. If no connections were made among the 3 genes (singlet interactions), a score of 1 was assigned. For comparison, 17 of the randomly-selected non-receptor genes were subjected to the same analysis with 2 nearby partners chosen, based on their proximity to the non-receptor gene.

To evaluate gene blocks for synteny, I used Ensembl to identify and locate established counterparts of the human receptor genes in other species, otherwise BLASTP was used to find a suitable ortholog/close homolog. By examining the chromosomal arrangement of some of the receptor genes across species, it was possible to gain additional insights into the evolution of receptor families and functional blocks of genes.

### 2.4. Evaluation of Gene–Gene Interactions

Gene–gene interactions among the receptor and non-receptor genes were evaluated with GeneMANIA [31] as described elsewhere [24]. Briefly, the lists of receptor and non-receptor genes were submitted for an analysis that was focused solely on gene interactions, as defined by the Lin et al. dataset [32]. No additional genes were allowed to be added in the construction of networks. The number of links per gene was obtained as a measure of connectivity. For comparison, 3 additional 30-member lists of randomly-selected genes (Supplementary Table S1) were prepared as described above, and were analyzed with GeneMANIA. These additional sets of random genes allowed for the establishment of a mean and standard deviation for the 4 lists of control genes, for statistical purposes.

To determine if functionally related genes in syntenic blocks interacted with one another in networks (as detected with GeneMANIA), I focused on a subset of receptor genes [13] identified as having undergone adaptive relocation during evolution (Supplementary Table S2). For this analysis, two genes in the vicinity were selected, based on their relationship (functional activity or close proximity) to the index gene. These neighboring genes are also listed in Supplementary Table S2. For comparison, 17 of the non-receptor genes were chosen, because they are known to form networks with other genes in GeneMANIA (part of the selection criteria discussed above). Two nearby genes were included in these 3-gene sets based on a similar proximity to the index non-receptor gene, as was observed for the receptor gene sets (listed in Supplementary Table S2). To be added to the 3-gene sets, the nearby genes had to participate in gene–gene interactions when tested on their own in GeneMANIA; otherwise, a lack of interaction with other genes would simply be due to their absence from the interaction dataset of Lin et al. [32]. For the GeneMANIA analysis, the 3-gene sets were evaluated individually to see if all three genes formed a network when the program was allowed to add 5 additional genes. 

### 2.5. Statistical Analysis

The average number of unique homologous matches obtained from BLASTP analysis and the standard deviation (SD) from the mean were determined by searching the human genome with the receptor and non-receptor protein sequences. The list of hits was inspected manually to derive the number of unique matches. A *t*-test was used to determine if differences comparing the receptor and non-receptor analyses were statistically significant at *p* < 0.05.

Likewise, a *t*-test was used to analyze differences between the blocks of receptor vs. non-receptor genes in terms of their connectivity scores based on GeneMANIA.

Gene interactions among the receptor and non-receptor lists of 30 genes were calculated as the number of links (connections) per gene from GeneMANIA. Data from 4 separate randomly-selected gene lists were averaged and analyzed to determine the mean and SD, as described previously [24]. This allowed for the establishment of a confidence interval that spanned three times the SD of the control data (*p* < 0.01).

## 3. Results

### 3.1. Summary of the Model and Supporting Evidence

**Figure 2:**
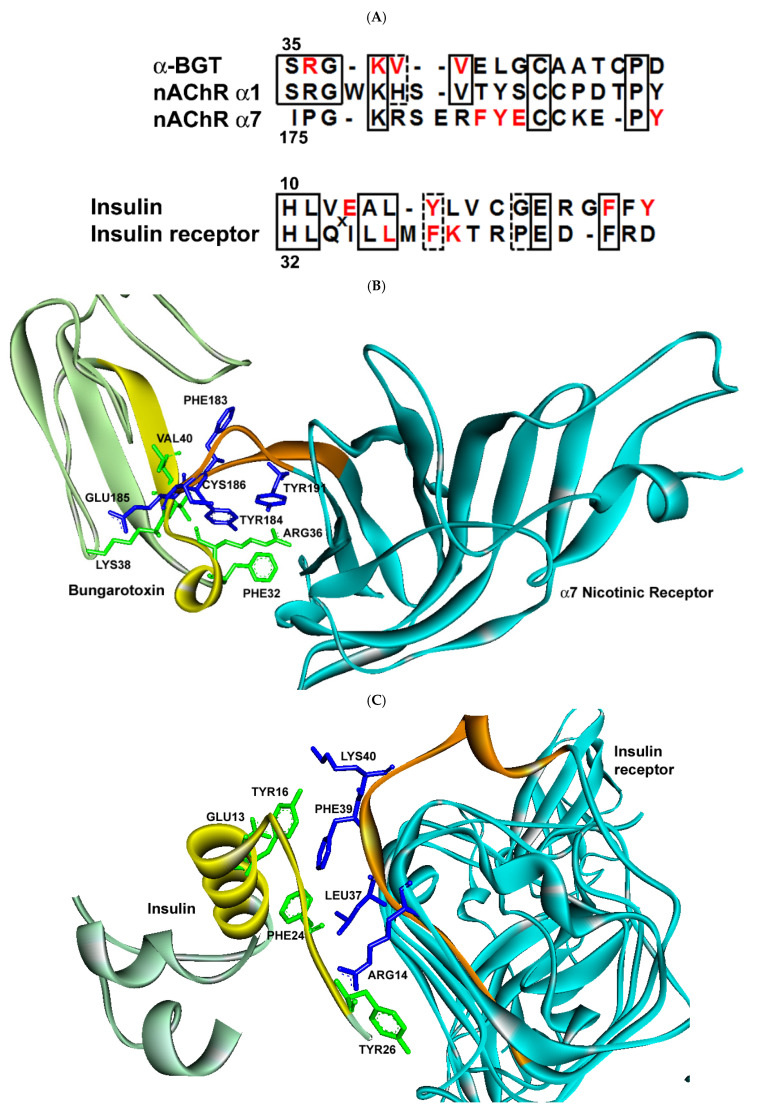
Self-binding peptide segments mediate binding to receptors. (**A**) The amino acid sequences of duplication units or regions of α-BGT and insulin were previously predicted to bind to their respective receptors (see refs. [1,3]) and are aligned with receptor sequences involved in binding. (Note: in the original discovery of regions of homology between α-BGT and the AChR [1], the α1 subunit sequence was used in the search.) The numbers refer to the locations of reference amino acids in the sequences associated with the three-dimensional structures [33,34]. Amino acids in the red font mediate binding, as seen in (**B**,**C**). (**B**) Co-crystal structure of α-BGT (light green ribbon and lime green side chains) and the α7 subunit of the nicotinic receptor (turquoise ribbon and dark blue side chains). The sequences in (**A**) have been highlighted in yellow in α-BGT and orange in the nAChR. Side chains involved in binding are displayed and numbered as in (**A**). (**C**) Cryo-EM structure of insulin (light green ribbon and lime green side chains) and the insulin receptor (turquoise ribbon and dark blue side chains) reveals amino acids involved in binding (numbered as in (**A**)). Again, the sequences depicted in (**A**) are highlighted in yellow (insulin) and orange (insulin receptor).

The general self-associating peptide model is summarized in Figure 1. The protective properties of the self-binding peptides toward the nucleotide sequences that encoded them favored the survival and duplication of these modules in the emerging genome. Some of the cohesion modules assembled into more complex domain structures that evolved into receptors, while the original self-binding module retained its ligand function. In the model, regions of ancient homology mediate ligand–receptor binding. Although this may not be true for more evolved pairs of ligand–receptor proteins, the predictive ability of the theory is significant. In 1989, based on the homologous interactions between ligands and receptors, I proposed that a specific amino acid sequence in α-bungarotoxin (α-BGT) should bind to a corresponding region in the nicotinic acetylcholine receptor [1] (Figure 2A). The co-crystal structure of α-BGT and the α7 nicotinic receptor reveals that these regions indeed form the major contacts that stabilize their binding [33], as predicted over 30 years ago (Figure 2B). Separately, I identified a duplication unit in insulin which is responsible for dimer formation [3] (Figure 2A), which, according to the model, should bind to the insulin receptor. In fact, this duplication unit (cohesion module) not only mediates dimer formation, it also binds to the receptor, as determined by cryogenic electron microscopy (EM) of the complex [34] (Figure 2C). In both cases, protein–protein binding involves a mixture of stabilization via aromatic rings (prominent), van der Waals forces, cation-pi orbital interactions, and charge complementation. It is quite remarkable that a simple analysis of sequence homology between ligands and receptors was able to accurately identify the regions that actually bind in three-dimensional space. 

### 3.2. Greater Number of Homologous Hits with Receptor Sequence Query

**Figure 3:**
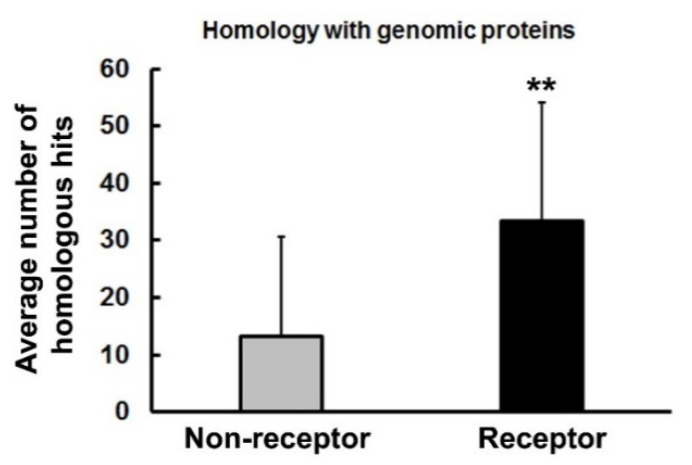
Comparison of BLASTP homology searches performed with 30 receptor and 30 non-receptor sequences. Unique matches with the query sequences were quantified and the data are expressed here as the average number of homologous hits in the genome for each protein analyzed. The error bars represent the standard deviations of the group data. Significant differences between groups are indicated with asterisks: ** *p* < 0.01.

The model in Figure 1 illustrates how self-binding peptides and cohesion modules were duplicated and widely dispersed in the genome. This process created a large pool of potential ligand–receptor pairs. Moreover, the nature of the corresponding genes—which are prone to insertion and duplication—led to propagation of very large families of genes, such that the immunoglobulin variable region family, histocompatibility locus antigens (HLA), GPCRs, and odorant receptor genes, which comprise a substantial fraction (>15%) of the total genome. Consequently, one would expect to find more examples of proteins in the modern genome with homology to receptors than to non-receptor gene products that did not experience these same selective pressures. This possibility was evaluated by searching for unique proteins with defined levels of homology to a representative set of receptor vs. non-receptor proteins that are listed in Supplementary Table S1. The receptor and non-receptor proteins were similar to each other in terms of their evolutionary conservation (77 and 67% conservation in *C. elegans*, respectively) and the percentage of essential genes in each list (35%). The results of the BLASTP searches are depicted in Figure 3. On average, each receptor showed homology (as judged by the sharing of identical and similar amino acid residues) with 33 other proteins in the genome (excluding the search sequence), whereas each non-receptor protein displayed homology to an average of 13 other proteins. Although these data were obtained with small subsets of proteins, there is strong reason to believe they will extrapolate to other receptor vs. non-receptor protein comparisons, because the subset of receptors was highly representative of this category of proteins. The homology hits that were detected in the BLASTP searches showed a range of similarity to the query sequences, with some matches being quite close and others more distant. The results are not exhaustively summarized in this section; instead, some representative examples are shown in Table 1 to provide a snapshot of the receptor data.

As can be seen in the table, NCAM1 is similar to other adhesion molecules (e.g., DCC, a netrin receptor), but also to extracellular matrix proteins, such as hemicentin-1. Homology between TENM4 and genomic proteins was largely mediated via its laminin and EGF domains. ITIH5 is interesting because it shows a high degree of homology with ITIH3, as expected, whereas its von Willebrand factor A-like domain showed weaker homology to sequences that were found in the CACNA2D4 calcium channel. Receptors are typically amalgams of unitary domains, such as immunoglobulin, fibronectin, and EGF domains. I propose that many of the regions of homology between receptors and other genomic proteins represent ancient dimer–heterodimer–assembly interaction sites, even though direct binding has not been demonstrated here. These specialized regions for association have been named cohesion modules, based on their similarities.

### 3.3. More Gene–Gene Interactions among Receptor Genes

**Figure 4:**
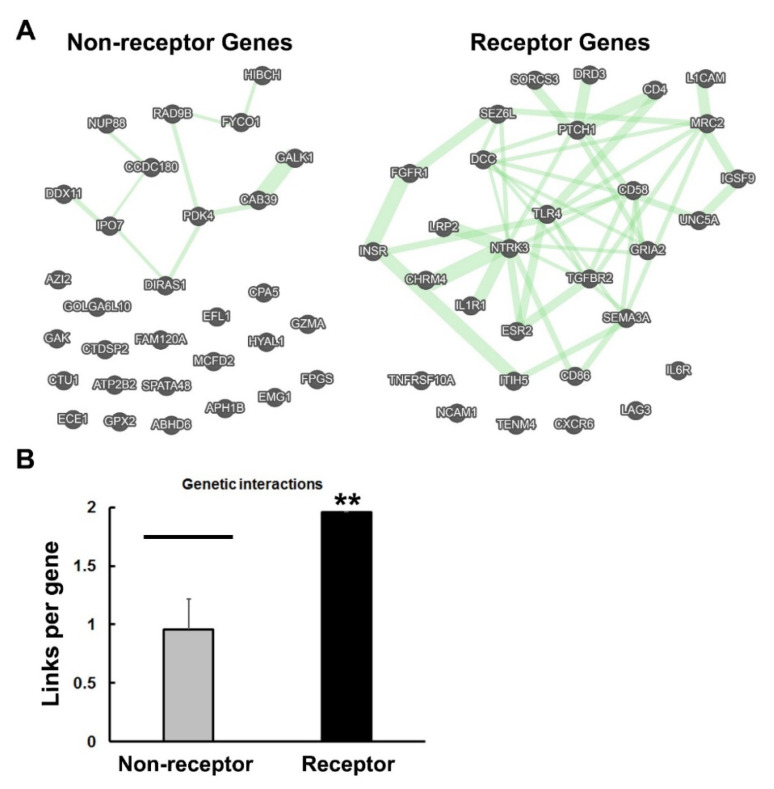
Comparison of genetic interactions among non-receptor and receptor genes. (**A**) The results of the GeneMANIA analysis are depicted with green lines indicating gene–gene interactions. The thickness of the green lines reflects the number of interactions in the dataset of Lin et al. [32] associated with the connected genes. The gene designations are shown and are compiled in Supplementary Table S1. The genes depicted at the bottom of the networks in (**A**) were ones that failed to connect with other members of the panel, despite the capability to interact with other genes in the genome, as determined by GeneMANIA. (**B**) The total number of links connecting the genes in each set was calculated automatically by GeneMANIA and the average links per gene was plotted. For comparison, four 30-member lists of randomly-selected genes (120 total) were analyzed in the same way, as described in the Methods section. This allowed for the calculation of a mean, SD and a 3 X SD confidence interval (black bar). Asterisks indicate significant differences with ** *p* < 0.01.

If ligands and receptors were derived from transposable elements, which included inverted repeats, these DNA sequences may have left remnants of their aptitude for self-binding, duplication, and assembly in the genome. When a series of inverted repeats was inserted and duplicated in the genome, these regions might be expected to interact with one another, due to their complementarity and similar folding. In addition, they may have survived hydrolysis and strand damage because of the protection conferred via these intra-strand interactions. If so, I hypothesized that the genomic regions that are associated with receptors should be more interactive, as manifested in greater involvement in gene–gene interactions. This possibility was tested by evaluating the genetic interactions of receptor vs. non-receptor genes in GeneMANIA. Networks generated from this analysis are shown in Figure 4A. Of the 30 genes in the lists, 24 were involved in genetic interactions among the receptor genes, whereas only 11 of the non-receptor genes were connected in this way. Furthermore, the receptor genes formed a significantly greater number of links per gene when compared with the randomly-selected non-receptor genes (Figure 4B). Clearly, this representative sample of receptor and non-receptor genes is distinguished by their propensities to interact with other genes of the same class. Previous studies have demonstrated that genetic interactions are a function of the degree of evolutionary conservation of genes, as well as their participation in essential functions [35]. Perhaps, the interactive properties of the receptor genes reflect their ancient origins, and a more extensive integration into networks of essential genes.

### 3.4. Identification of Syntenic Blocks Containing Receptor Genes

**Figure 5:**
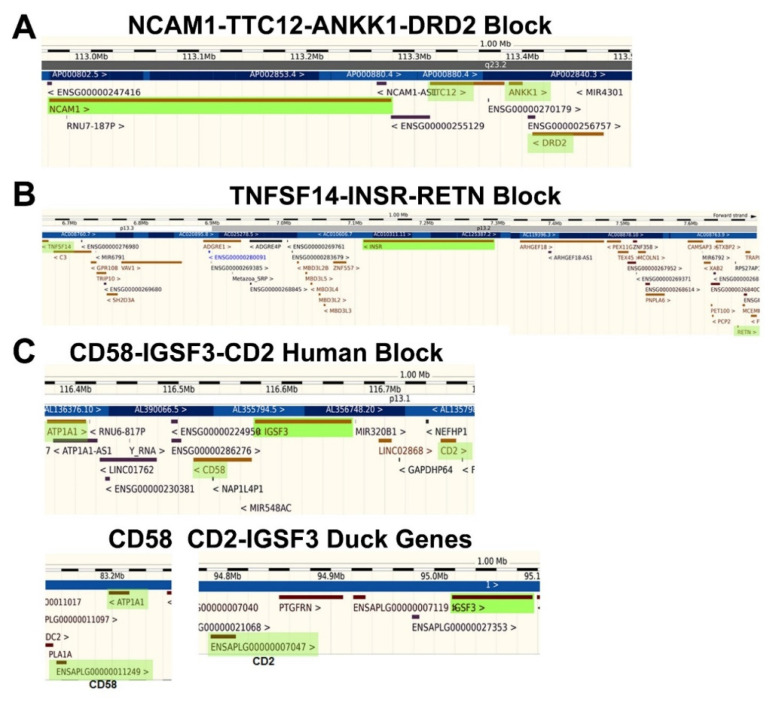
Syntenic blocks of receptor genes. (**A**) NCAM1 was selected for this study and happens to lie in a functional block described previously [30]. (**B**) INSR is located in a second block (highlighted in green in the center). It is flanked on the left by TNFSF14 and on the right by RETN (both highlighted in green). (**C**) CD58, IGSF3 and CD2 lie in close proximity in the human genome. In contrast, there is a gap of 11.6 Mb between CD58 and CD2 in the duck genome, and CD2 is slightly farther away from IGSF3. ATP1A1 has been included as a shared location marker.

Functional blocks of genes that formed during evolution have been described previously [25,26,27], and include clusters of genes with significance for medical conditions [23,24,28]. The rearrangement of these genes into syntenic blocks has been referred to as adaptive gene relocation [29] or gene amalgamation [23]. When the local genomic environment of some of the receptor genes was examined in greater detail, it appeared that a subset of these genes may reside in syntenic blocks. Briefly, the criteria include the co-localization of the genes on human chromosomes, with evidence of greater contiguity than in other species, and involvement of the block of genes in common biological functions [24]. Interestingly, the NCAM1-TTC12-ANKK1-DRD2 functional gene cluster (depicted in Figure 5A) was already recognized in earlier studies [30]. A second example is the tumor necrosis factor superfamily 14 (TNFSF14)-insulin receptor (INSR)-resistin (RETN) block on chromosome 19 (Figure 5B). These genes are collectively involved in the regulation of energy metabolism and insulin responsiveness [36,37,38]. In mice, INSR and RETN are nearby each other on chromosome 8, whereas TNFSF14 is on chromosome 17. In contrast, RETN is on the same chromosome as TNFSF14 in the green anole lizard, although they are far apart, whereas INSR is on a different chromosome. Overall, comparative genomics revealed that these three genes are closest in the human genome.

The final example is the CD58-IGSF3-CD2 gene block on chromosome 1 (Figure 5C). For comparison, the arrangement of the corresponding duck genes has been included to illustrate the convergence of functionally-related genes on a compact location in the human genome, which might allow for the joint regulation of their expression. CD2 and CD58 are homologous to each other and are related by a gene duplication event [39]. They are part of the larger CD2/CD48 family of immunoglobulin related receptors, which are found on lymphocytes [40,41]. CD2 and CD58 are near to each other in zebrafish [41], but far away from IGSF3 on the same chromosome, whereas in canaries, CD2 resides close to IGSF3, but distant to CD58. Gene rearrangements in humans have brought these three genes into their closest observed proximity.

### 3.5. Increase in Genetic Interactions among Members of Receptor Gene Blocks

**Figure 6:**
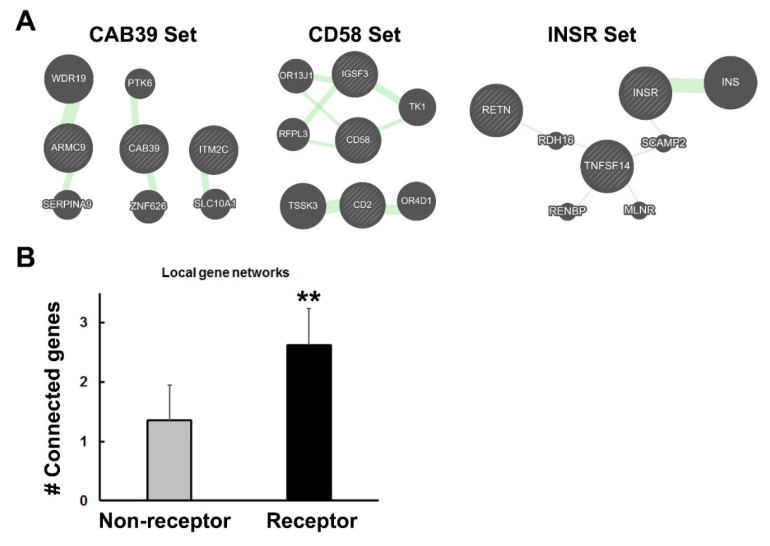
Analysis of gene-set genetic interactions. (**A**) Several 3-gene sets are depicted here as examples of the analysis. They were evaluated with GeneMANIA to determine if they connected into networks, as described in the Methods section. The thickness of the green lines and size of the added network genes (indicated by circles with solid fill) reflect the number of interactions associated with that pair as determined by GeneMANIA. On the left, a non-receptor gene, CAB39 and two nearby genes, ARMC9 and ITMC2 were tested. Although they connected to other genes, they did not connect to one another, even indirectly. This set received a score of 1 for singlet interactions. In the middle, the CD58 3-gene set showed indirect connections between CD58 and IGSF3 but not to CD2. This set was scored as a 2. At the right, the INSR set showed that all three genes connected with each other indirectly through intermediates. This set received a score of 3 for statistical analysis. (**B**) The number of connected genes for the 3-gene sets was calculated for non-receptor and receptor gene blocks (Supplementary Table S2). A *t*-test revealed significantly more connections among the receptor panel of genes with *p* < 0.01 **.

If some receptor genes have been rearranged and selected for their proximity to related genes, one might expect to observe that the local gene environment may show a greater connectivity, as judged by increased interactions involving nearby genes. These interactions may reflect linkage disequilibrium, residence in topologically associated domains, or epistasis, among other connections. The focus of this study was restricted to those genes that reside in syntenic blocks, which appear to be a small subset of genes, but which have significant medical implications [23,24,28,30]. To test for local interactions, I created sets of three genes—the original receptor or random non-receptor gene plus two additional genes in the immediate vicinity of the index gene. The goal was to determine if the three genes were connected to each other (directly or indirectly) when a small number of other genes (five) were added by GeneMANIA to construct interaction networks. Examples of this analysis are depicted in Figure 6A. The numbers of genes from the three-gene sets that interacted with each other were tabulated, as described in the Figure legend. As seen in Figure 6B, the receptor genes formed networks, with significantly more members of the three-gene sets connected to each other than was observed for the random (control) gene sets. These data support the findings above regarding increased genetic interactions among the representative set of receptor genes in comparison to the randomly-selected non-receptor genes. Moreover, they extend this observation to include interactions with nearby genes that were selected for their proximity or functional relationships, and not just fellow genes from the receptor class.

## 4. Discussion

The evolution of peptide/protein ligands and their receptors can never be fully unraveled; however, important clues have emerged by examining the genomic traces of this process. Here, I sought to further explore receptor evolution by investigating whether receptor proteins and their genes exhibited properties that are expected from the self-associating peptide model, aka the trexon theory [3]. As predicted, homology searches with a representative sampling of receptor amino acid sequences detected a significantly larger number of homologous matches from the genome when compared to searches with non-receptor sequences. Of course, hits from the same family of receptors were identified, but additional proteins with more remote relationships were also found. In addition, the corresponding genes that encoded the receptors were more interactive than for the non-receptor genes, both with other members of the receptor list and with genes that were located nearby on the chromosome. Finally, in some cases, the receptor genes were clearly arranged in syntenic blocks of genes geared toward a common purpose, such as immune stimulation (CD2-CD58), energy metabolism (INSR), or neurotransmission (NCAM1-DRD2), as discussed below.

According to the extended model presented here, the greater homology between receptor proteins and genomic proteins when compared to non-receptor proteins reflects the preservation of cohesion modules in the genome. It is worth noting that the homologous regions in receptors may span more than a single defined domain, such as in the immunoglobulin and fibronectin domains. The core cohesion modules would have predated the evolution of more specific ligand–receptor interactions and were based initially on homologous interactions [1,3] with the incorporation of complementarity as evolution progressed [4,7]. Evidence of this process is shown in Figure 2, where binding between homologous sequences now takes advantage of complementary charge interactions, in addition to van der Waals and aromatic interactions that are mediated by chemically similar amino acids. Evolution has served as a locksmith to mold and refine more familiar lock-and-key binding arrangements from cohesion modules, which initially interacted based solely on their similarity. Binding that is mediated via homologous regions of proteins may reflect either divergent or convergent evolution of cohesion modules, as suggested earlier [1]. As proposed in the model, cohesion modules broadly mediated dimer formation, subunit assembly of similar components, and assorted protein–protein interactions, mostly of lower affinity [9]. As their name implies, the cohesion modules would have shown mutual attraction through cohesive forces or ‘like binds like’ via hydrogen bond networks, as in water, or dispersion forces, as in micelle/membrane formation [1]. The significance of these putative modules is highlighted by the presence of a large number of relics (duplicated ‘copies’) in the genome. More recent studies [42] confirmed my much earlier findings [1] of protein self-association being a reflection of evolutionary processes. 

In some cases, the receptor genes appear to have been rearranged into blocks of functionally-related genes. Syntenic clusters of genes with a common purpose have been described previously [23,24,25,26,27,28] and include integrons in bacteria [25], a block contributing to cardiomyopathy in humans [28], and the NTAD cluster (NCAM1-TTC12-ANKK1-DRD2 [30]), mentioned above as part of this study. The NTAD block of genes jointly affects dopaminergic neurotransmission and neuronal development [30]. This cluster was identified on the basis of single nucleotide polymorphisms in the region that enhances the risk for psychiatric disorders [43]. One key example from the present work is the CD2-CD58-IGSF3 gene block. CD2 and CD58 are homologs, as pointed out by Seed [39]. Because they contain immunoglobulin domains, they also resemble IGSF3, although this is a more distant relationship. CD2 and CD58 interact with each other as part of their normal recognition/signaling function in the immune system [44]. This is a clear confirming example of the self-associating peptide theory. CD4-LAG3 is another pair of homologous receptors that are located adjacently on chromosome 12, and are functionally related to one another [45]. CD4 and LAG3 up- and down-regulate T cell activation [46], respectively, through their interactions with class II major histocompatibility antigens. The protein domains that mediate these various interactions represent the evolved counterparts of original cohesion modules.

Additional examples of functionally-related blocks of genes include CCR9 and IL1R2 chromosomal regions, where chemokine receptors or cytokine receptors have been duplicated and retained in clusters. Interestingly, another receptor block identified here includes INSR-TNFSF14-RETN. TNFSF14 and resistin (RETN) participate in the regulation of insulin responsiveness [36,37,38], but tend to oppose its actions. It is not known whether these proteins interact at any level, but there is a functional complementarity in their actions that is similar to the insulin–glucagon story developed by Root-Bernstein and colleagues [4,47]. Accordingly, this block of genes may represent an example of peptide (and biological) complementarity that was facilitated by gene amalgamation and subsequent selection. The syntenic gene blocks described here may have persisted due to their location in recombination coldspots, via the deletion of insertion/mutational hotspots, or through other mechanisms that prevented their disruption.

The select set of representative receptor genes that was examined in this study was also characterized by a greater genetic connectivity among these genes than was observed for a similar-sized set of non-receptor genes. The genetic interactions detected in this study were based on GeneMANIA and the Lin et al. [32] dataset, and are likely to be a complex mixture. This might include local interactions within DNA domains, long-range physical interactions and epistasis, i.e., when the effect of variation in a gene depends upon or is modified by one or more independent background genes. Greenspan [48] proposed that genes are naturally interactive and distribute into networks, which promotes flexibility in the genome. Moreover, the propensity for genes to interact appears to depend, in part, upon their degree of evolutionary conservation [35]. Therefore, older genes, such as the receptor genes and their trexon building blocks, may be more fully integrated into functional gene networks. In addition to the increase in genetic interactions when compared to non-receptor controls, the receptor genes were more connected in gene networks with nearby and related genes in the three-gene sets. This was not a guaranteed outcome, and suggests a special relationship between some receptor genes and their local chromosomal environments. I speculate that formation of these gene blocks might allow for the joint regulation of gene expression, although this was not demonstrated here. Joint regulation of gene expression is especially important when the timing and tissue co-expression of genes are critical to development and/or function; co-localization may have achieved the tight coordination of gene activity. The interactive nature of the receptor genes may reveal a form of ‘molecular memory’ of their initial creation via their duplication, transposition, and amalgamation into the genome [49]. Signature features of DNA sequence/structure that once conferred mobility and replicability on gene segments may today serve as organizing landmarks in the genome.

In a compelling example of symmetry in nature, networks of genes appear to have created networks of proteins that interact because of cohesion. How was this accomplished? The actual mechanisms will remain shrouded in uncertainty; however, tantalizing clues do exist. The trexon theory proposed that self-binding nucleotides (inverted repeats) coded for self-binding peptides. Moreover, it was suggested that self-binding peptides that bound to the nucleotide sequences encoding them were preferentially selected because they protected the nucleotide polymers against hydrolysis [3] (see Figure 1); there is evidence to support this idea [14]. These interactions presuppose crosstalk between nucleotide and amino acid sequences during evolution. Blalock et al. and others observed that the amino acids encoded by the sense and antisense codons have a complementary relationship to one another [15,50]. This relationship has been exploited to generate sense and antisense peptides that physically interact [16], although the generality of this phenomenon is questionable [7]. Regardless of its utility, the relationship between codons, antisense codons, and the properties of the amino acids they specify is non-random and suggests a dynamic and mutual co-evolution. Chemical insights into this co-evolution suggest that RNAs detect the polarity of amino acid side chains in relation to codon and anticodon sequences [51], which would start to explain why antisense peptides sometimes interact with the sense-coded peptide. Cohesive forces likely drove this relationship between codons and amino acids. According to the model, cohesion modules in proteins mediate protein–protein interactions, and cohesion domains in the genome mediate local and long-range DNA interactions. It is possible that the cohesion domains in DNA that are proposed here overlap with genomic regions that form intra- and inter-chromosomal contacts, including topologically associated domains [52,53] (see Figure 1). DNA cohesion domains may have facilitated adaptive gene relocation to form syntenic blocks of genes and, more broadly, the search for homologous regions during recombination, repair, and transposition [54]. 

Retrospective studies of evolutionary processes face certain limitations or alternative interpretations of the findings. First, it is challenging to attempt to peer into the past to explain the state of the genome and protein–receptor interactions that are observed today. Second, receptor and non-receptor proteins were evaluated here for homology with other proteins in the genome according to the same criteria; however, there was not a methodical attempt to control for protein size. Nevertheless, the two sets of proteins generally showed a similar distribution of protein sizes, so this is unlikely to have biased the results. The homology that was detected is not intended to imply that the homologous proteins were ancestral to each other, or that they will necessarily interact physically. Instead, the homology can be viewed as a surrogate indicator of cohesion modules that bear a resemblance because these regions mediated important functions during evolution, such as protein–protein interactions, protein–DNA/RNA interactions, the assembly of subunits, etc. A genome-wide search for cohesion module homologs was not conducted, owing to the large expanse of the genome that is devoted to receptor genes. However, the expanded array of receptors in the genome only serves to support the findings generated here with a limited, but representative, set of receptor genes. The subset of receptor genes clearly participated in more gene–gene interactions than a subset of random genes, as judged by their interactions with other receptor genes and with neighboring genes on the chromosome. These genetic interactions were identified in GeneMANIA based on the data of Lin et al. [32]. Alternative measures of gene interactions—chromatin mapping, their occurrence in gene factories, and their shared transcriptional regulation—may have generated a different pattern of results. Nevertheless, GeneMANIA has previously proven useful for analyzing genetic interactions among disease risk genes [24,35]. Finally, evidence was provided to support the discovery of syntenic blocks of receptor genes; however, they will need further experimental verification of their functional connections. 

In an evolutionary environment characterized by harsh initial conditions, early chemical species, peptides, and other polymers that survived and replicated must have displayed special properties and/or evolved ‘strategies’/adaptations that resisted chaotic destruction. Self-organization via cohesion—similar molecules literally sticking together for survival—appears to have been an effective adaptation that emerged [55]. As evolution progressed, the association of dissimilar, but complementary, molecules (such as nucleotides) with peptides/proteins probably provided the broader survival of chemical species, which may have then been adapted for other forms of small molecule ligand–receptor interactions. In this way, a simple driving force—self-association to avoid destruction—selected for the emergence of cohesion modules (in proteins and DNA) that mediated the evolution of specialized binding proteins (receptors) and the interactive genes that encoded them. Future studies may provide additional support for the validity of this model or stimulate the development of alternatives.

## Figures and Tables

**Table 1:** Representative hits from screening for homology with receptors and domain matches *.

Query Protein	Homologous Hits	Homology (*E* Value)	Domain Match
NCAM1	Hemicentin-1	4e^−41^	Ig
	DCC netrin receptor	4e^−29^	Ig, Fn
	Titin	6e^−29^	Ig
TENM4	Tenascin XB	4e^−45^	Laminin, EGF
	Tenascin R	3e^−22^	Laminin, EGF
	Integrin subunit β-4	6e^−12^	Laminin
ESR2	Retinoid X receptor γ	8e^−49^	NR-DBD, NR-LBD
	Androgen receptor	5e^−26^	NR-DBD, NR-LBD
	Vitamin D receptor	5e^−18^	NR-DBD
ITIH5	Inter-alpha-trypsin inhibitor heavy chain-3	8e^−121^	ITIH heavy chain
	Von Willebrand factor A domain containing-3A	7e^−7^	vWFA-like
	Ca^++^ voltage-gated channel subunit α2δ4	0.001	vWFA-like

* The following abbreviations appear in the Table: Ig, immunoglobulin; Fn, fibronectin; EGF, epidermal growth factor; NR-DBD, nuclear receptor DNA-binding domain; NR-LBD, nuclear receptor ligand-binding domain; ITIH, inter-alpha-trypsin inhibitor; and vWFA-like, von Willebrand factor A-like domain. Amino acid sequence homology was evaluated with BLASTP. Only a few representative examples are shown here for each query protein, including representative protein domains, although homology was not restricted to established domains.

## Data Availability

Data related to this paper are included in the main and Supplementary Materials.

## Supplementary Materials

The following are available online at https://www.mdpi.com/article/10.3390/life11121335/s1, Supplementary Table S1: List of Receptor and Non-receptor Genes; Supplementary Table S2: Receptor and Non-receptor Gene Sets.
